# Dementia-Friendly America Recognition: Strategies for Urban, African American Communities

**DOI:** 10.1093/geroni/igab046.1029

**Published:** 2021-12-17

**Authors:** Tarisha Washington, Susan Frick, Raj Shah

**Affiliations:** 1 Rush University Medical Center, Chicago, Illinois, United States; 2 Rush, Rush University Medical Center, Illinois, United States

## Abstract

Dementia-Friendly America is a network of communities across the United States who have committed to a process to support people living with dementia and their caregivers. Through technical support from Dementia Friendly Illinois, CATCH-ON, a HRSA Geriatric Workforce Engagement Program, has identified key characteristics for the 17 communities in Illinois achieving national recognition and for communities that have engaged but not yet achieved national recognition. In addition to communities in rural regions, urban communities with a large number of African Americans residents have necessitated more grassroots engagement than other communities. Partnerships are vital for providing information and education about the movement and for supporting multi-sectoral engagement. This presentation highlights barriers and facilitators in diverse communities, particularly urban African American communities, becoming recognized by Dementia Friendly America.

